# Situational influences on officiating performance: An analysis of foul calls in the Chinese basketball association league (CBA)

**DOI:** 10.1371/journal.pone.0342151

**Published:** 2026-02-19

**Authors:** Fang Shu, Liqing Zhang, Xiao Xu, Ruohan Xu

**Affiliations:** 1 Sports Coaching College, Beijing Sport University, Beijing, China; 2 College of Physical Education, Dalian University, Dalian, China; 3 School of Journalism and Communication, Beijing Sport University, Beijing, China; NED University of Engineering and Technology, PAKISTAN

## Abstract

The growing emphasis on situational variables has significantly advanced research in sports performance analysis, yet studies focusing on referees from this perspective remain limited. This study examines how multiple situational variables influence foul calls metrics during the 2022–2023 Chinese Basketball Association League (CBA) season. Drawing on 18,519 foul events from 441 games, with the final analytical sample comprising 18,182 events from 439 games, independent-sample t-tests were used to assess the effects of individual situational variables, and binary logistic regression models were applied to examine their associations. The situational variables analyzed included game system, game type, team quality difference, score differential, and foul differential. Results show that while a clear home advantage emerges in home-and-away games, the overall foul number does not differ across systems. Stronger teams were called more fouls in games with large team quality gap, particularly in the fourth quarter. Leading teams were consistently called for more fouls when score differential widened, and teams with fewer accumulated fouls were more likely to receive subsequent calls, suggesting a potential balancing tendency in officiating. Logistic regression showed that foul calls was significantly associated with score differential, foul differential, and team quality difference, whereas the main effects of game type were limited. Importantly, several variables that were non-significant in single-factor tests emerged as significant in the multivariable models, underscoring the situational nature of officiating. This study enriches understanding of officiating performance within the framework of sports performance analysis. These findings highlight that referees’ decisions are “context-sensitive” rather than random or rigid, meaning they vary across specific competitive situations. Such situational sensitivity may sustain competitive balance and game flow but also introduces risks of officiating bias. Recognizing this dual nature is valuable not only for referee evaluation, but also for leagues governance, as well as for teams seeking to anticipate officiating tendencies during games.

## 1. Introduction

Referees are indispensable participants in basketball games. Much like players, their decisions directly shape game dynamics and can influence final outcomes. The influence of referees derives from both the inherent complexity of basketball and the discretionary nature of officiating [1–3]. In certain situations, officiating decisions may become turning points within games and focal points in public debate afterwards. Empirical evidence has demonstrated that specific officiating interventions—such as technical fouls and disqualifications—can significantly affect game results [4,5]. In addition, free-throw opportunities are frequently incorporated into post-game analyses as indicators of strategic and tactical significance [6,7]. Accordingly, the central question of this study is how multiple situational variables, when considered simultaneously, are associated with foul-calling patterns in professional basketball. Using play-by-play data from the Chinese Basketball Association league (CBA), we examine both single-variable effects and multivariable associations to provide an integrated account of situational influences on officiating performance.

Sports performance analysis provides systematic and scientific approaches to observing and evaluating the performance of athletes, teams, and other participants. Although basketball performance analysis has traditionally focused on players and teams, the increasing transparency brought by the publication of officiating reports has extended analytical interest to referees [8]. Within this framework, officiating can be conceptualized as a form of performance that can be examined using similar principles and methods to those applied to players and teams.

Early research on officiating performance predominantly emerged from investigations of home advantage, a pervasive phenomenon in professional sport. In basketball, the number of fouls called has frequently been treated as a key indicator of such advantage [9]. Subsequent studies examined officiating behavior across different contexts and discussed various forms of referee bias. For example, Nevill and Holder suggested that referee bias may partly explain home advantage, whereas Thu et al. reported that referees display systematic tendencies in nationally televised games, particularly when score differentials are large [10,11]. Using NCAA data, Anderson and Pierce further demonstrated that foul calls are influenced by foul differentials and score differentials [12]. Price et al. introduced the distinction between discretionary (DTO) and non-discretionary (NTO) calls, and identified bias about home teams, trailing teams, and teams behind in playoff series [13]. More recent work has explored the effects of flopping, offensive fouls, and technical fouls on subsequent calls, as well as the role of video review in evaluating officiating accuracy [14–17]. Collectively, this body of research indicates that officiating performance is systematically associated with specific situational variables, such as home–away status, score differentials, and accumulated foul differentials. However, because these factors are most often examined in isolation, it remains unclear how multiple situational variables operate concurrently within the same game context. This limitation underscores the need for an integrated analytical framework that considers multiple situational dimensions simultaneously when examining the foul-calling pattern.

Within the broader framework of sports performance analysis, situational variables are widely recognized as critical determinants of game dynamics. Variables such as game systems, game type, team quality, and game conditions are essential for interpreting tactical choices and evaluating player performance [18–21]. Prior research has tended to examine single situational variables in isolation, leaving the concurrent consideration of multiple situational variables within the same game context largely unexplored.

Building on this gap, the present study investigates how multiple situational variables jointly affect officiating decisions in professional basketball. Using data from the CBA 2022–2023 season—which provides a quasi-experimental context due to the coexistence of centralized non–home-and-away and traditional home-and-away competition systems under COVID-19–related constraints [22]—this setting offers a distinct analytical advantage. Specifically, the presence of two game systems within the same league and season allows comparisons of officiating behavior across different competitive formats while holding constant league rules, officiating standards, team composition, and seasonal conditions. This within-season, within-league variation reduces institutional and contextual confounding that often affects cross-league or cross-season analyses, thereby strengthening the internal validity of situational effect assessments. We analyze the effects of game systems, game type, team quality difference, and game conditions on foul calls by first examining the independent influence of each situational variable. We then incorporate all variables into unified binary logistic regression models to assess their combined associations under shared game contexts, thereby identifying which variables most strongly shape foul calls without specifying explicit interaction terms.

Guided by previous research and the specific focus of this study, we propose the following exploratory hypotheses. First, foul calls are expected to vary across individual situational variables: foul frequencies may differ between game systems and between home and away teams, between regular-season and playoff games, and across different levels of team quality difference. In addition, foul calls are expected to vary under different game conditions, including varying score and foul differentials. These hypotheses address the independent associations of each situational variable. Second, beyond these single-variable influences, we further hypothesize, in an exploratory manner, that when multiple situational variables are considered simultaneously, their combined associations with foul calls may reveal situational influences that are not captured by isolated comparisons.

Despite extensive research on home advantage and specific forms of referee bias, foul calls have rarely been examined within a single, integrated situational framework using event-level data from professional leagues. By jointly modeling game systems, game type, team quality difference, score differential, and foul differential within the same analytical framework, the present study provides a concise and comprehensive account of how situational variables are associated with officiating performance in professional basketball.

This study contributes to existing knowledge by applying a quantitative, sports performance analysis-based methodology to officiating research. Rather than centering on the question of “referee bias,” we focus on situational variables of officiating games. The findings enrich understanding of the role of situational variables in basketball officiating and contribute to a more nuanced account of how contextual conditions influence referees’ decision making.

## 2. Methods

### 2.1. Dataset

The dataset comprised foul calls and associated situational information from 441 games contested by 20 teams during the CBA 2022–2023 season. All data were obtained from the official CBA statistics website (www.cbaleague.com). Data extraction was conducted for research purposes between April and June 2024. Only publicly available, aggregated match statistics were used, no identifiable personal information about referees or players was accessed. Foul calls events and their corresponding contextual information were systematically collected, organized, and merged into the dataset. Because referee appointments vary across seasons in the CBA, restricting the sample to a single season helped ensure greater consistency in officiating standards and league policies. This design choice improves internal consistency and reduces cross-season institutional variation, but it also limits the generalizability of the findings to other seasons. Accordingly, the present analysis prioritizes standardized situational comparisons within a single competitive context, while future research could extend this framework to multi-season data to assess robustness.

After data collection, a series of cleaning procedures were applied to enhance the reliability of the dataset. Games with excessively large score or foul differentials may produce abnormal competitive conditions, under which foul calls are not representative of typical officiating performance. Such games are also relatively rare, meaning that their foul data provide limited inferential value. To minimize the influence of these outliers, foul-call events associated with extreme score or foul differentials were excluded using the three-sigma (3σ) rule, a commonly applied statistical criterion in performance analysis to improve robustness of inference [23]. This trimming was conducted at the event level and applied within situational sub-samples defined by non-zero score or foul differentials, rather than by removing entire games. In addition, two games that were officially declared invalid by the CBA were removed.

Technical fouls and disqualifying fouls were excluded. These calls accounted for 337 of the 18,519 initial foul events (1.82%) and typically arose from non-contact incidents, off-court situations, or violent behavior. Because they primarily reflect disciplinary sanctions rather than normal competitive interactions, they were deemed unsuitable for evaluating overall officiating tendencies in general game situations. Furthermore, overtime periods were excluded because they occur infrequently and vary in length, resulting in limited and inconsistent foul data. Accordingly, only foul calls from regulation time (four quarters) were analyzed, and the final analytical sample retained only common personal fouls and unsportsmanlike fouls occurring during regular on-court play.

Following these cleaning and exclusion procedures, the final dataset comprised 18,182 valid foul calls records (98.18% of the original sample) from 439 games (99.55% of all recorded games). Each foul event retained key situational variables, including game systems, game type, participating teams, game quarter and real-time score at the moment of the call. These situational variables served as explanatory factors, while the called team served as the response variable in the subsequent analyses.

### 2.2. Variables

In the regression models, the dependent variable was the team on which the foul was called under specific game situations, specified in three comparative contexts: home versus away teams, leading versus trailing teams, and teams with more versus fewer accumulated fouls.

The situational variables examined in this study included: Game systems (XFmt): non-home-and-away (neutral, closed venues without spectators) vs. home-and-away (with designated home and away courts). Game type (XTyp): regular season vs. playoffs. Team quality difference (XQD): based on teams’ win percentages in the 2022–2023 season. Using K-means clustering, all 20 teams were classified into strong, medium, and weak categories (coded as 3, 2, and 1), and the differential in team codes between opponents was used to represent game quality difference (large difference, small difference, evenly matched)[24,25]. In the CBA, team rankings are determined by cumulative win–loss points, and ranking-based classifications may separate teams with similar win percentages due to marginal differences in ranking order. Accordingly, K-means clustering was adopted to derive team quality categories by minimizing within-group variability while maximizing between-group differences. Score differential (XSD): real-time score margin between the two teams at the moment of the foul call. Foul differential (XFDG, XFDQ): cumulative foul differential between the two teams at the moment of the call, calculated at the game level (XFDG) and quarter level (XFDQ). Quarter (XQtr): the game period in which the foul occurred (Q1, Q2, Q3, or Q4). For statistical modeling, the situational variables were coded as follows: Game system (XFmt): 1 = home-and-away, 0 = non–home-and-away; Game type (XTyp): 1 = regular season, 0 = playoffs; Team quality difference (XQD): 2 = large difference, 1 = small difference, 0 = evenly matched; Quarter (XQtr): 1 = Q1, 2 = Q2, 3 = Q3, 4 = Q4; Score differential (XSD) and foul differential (XFDG, XFDQ) were entered into the analyses as continuous variables in their original scales, with no transformation or centering applied.

These situational variables were used both in single-factor analyses and as explanatory variables in multivariate models, allowing us to examine how they influence foul calls across different game contexts.

### 2.3. Data analysis

Statistical analyses were conducted using SPSS 20.0 (IBM, Armonk, NY, USA). First, independent-sample t-tests were applied to examine differences in foul calls across single situational factors, including game system, game type, and team quality difference categories. Due to COVID-19-related restrictions in the 2022–2023 season, games were played under two competition formats: centralized non–home-and-away (n = 278) and traditional home-and-away (n = 161). Home-win rates under the two game systems were descriptively compared to provide contextual information about differences between centralized non-home-and-away and home-and-away formats, and were not intended as inferential evidence of home advantage or officiating effects [26–29]. For team quality difference, all t-tests compared foul calls on the stronger team versus the weaker team within each differential category, thereby preserving the directionality of team strength in the analysis (for evenly matched games, team comparisons were conducted between the home and away teams, as no directional quality distinction existed). For game conditions variables, proportion and trend analyses were used to compare foul calls distributions difference between leading vs. trailing teams and between teams with more vs. fewer fouls, under varying score differential and foul differential (game-level and quarter-level).

Single-factor analyses were first conducted to provide descriptive and preliminary insights into the associations between individual situational variables and foul calls. To assess the combined influence of multiple situational variables, binary logistic regression models were constructed with game system (XFmt), game type (XTyp), quarter (XQtr), team quality difference (XQD), score difference (XSD), and foul differences (XFDG, XFDQ) as explanatory variables. The dependent variable was the called team in three comparative contexts: home vs. away, leading vs. trailing, and more vs. fewer fouls. Although explicit interaction terms were not included in the logistic regression model, this multivariate framework enables examination of the combined contextual influences of multiple situational variables on officiating performance [12,30]. In this context, “combined influence” denotes the simultaneous estimation of multiple situational variables within a single model, allowing their independent associations to be evaluated under multivariate control, rather than the testing of interaction or synergistic effects between variables. This design aligns with the study’s aim of exploring integrated situational effects. For logistic regression, the foul call outcome was coded as a binary variable in three comparative contexts: Home vs. away: 1 = foul called on the home team, 0 = foul on the away team. Leading vs. trailing: 1 = foul called on the leading team, 0 = foul called on the trailing team. More vs. fewer fouls: 1 = foul on the team with more accumulated fouls, 0 = foul on the team with fewer fouls. The model setup is as follows:


$$In\frac{P_{i}}{\text{1}-P_{i}}=\beta_{\text{0}}+\beta_{\text{1}}X_{Fmt}+\beta_{\text{2}}X_{Typ}+\beta_{\text{3}}X_{Qtr}+\beta_{\text{4}}X_{QD}+\beta_{\text{5}}X_{SD}+\beta_{\text{6}}X_{FDG}+\beta_{\text{6}}X_{FDQ}$$

Independent sample t-test was selected because it is suitable for comparing group means across categorical factors, while binary logistic regression was employed to model the likelihood of foul calls against specific teams under varying situational conditions, making it appropriate for binary officiating outcomes. Proportion and trend analyses were used to describe foul calls distributions difference under different score and foul differentials, thereby identifying status-based foul calls tendencies. The logistic regression models were used for explanatory rather than predictive purposes, and the estimated odds ratios (ORs) were interpreted as indicators of directional tendencies in foul calling. Specifically, ORs greater than 1 indicate a higher likelihood of a foul being called on the team coded as 1, ORs less than 1 indicate a lower likelihood, and an OR equal to 1 indicates no difference in foul-calling tendency between the compared teams. Table 1 summarizes the classifications of situational variable and the corresponding statistical methods used in the analysis.

**Table 1:** Classifications of situational variables and statistical methods.

Situational Variable	Classifications	Statistical Test/ Model	Comparison
Game systems	Non-home-and-away/ Home-and-away	t-test	Home team vs. Away team
Game type	Regular season/ Playoffs	t-test	Regular season vs. Playoffs
Game quality difference	Large/Small difference/ Evenly matched	t-test	Large & Small difference: Stronger vs. Weaker; Evenly matched: Home vs. Away
Game conditions	Leading vs. Trailing/ More fouls vs. Fewer fouls	Proportion and trend calculation	Leading teams vs. Trailing teams; Teams with more foul vs. Team with fewer foul
Quarters	Q1/ Q2/ Q3/ Q4	Binary logistic regression	Quarters included as model covariate
Comprehensive Variables	Multiple explanatory variables	Binary logistic regression	Home vs. Away; Leading vs. Trailing; Teams with more foul vs. Team with fewer foul

## 3. Results

### 3.1. Comparative analysis of foul calls under different situations

To address Hypothesis 1, this section presents a comparative analysis of foul calls across individual situational variables and game conditions, including game systems, game type, team quality difference, and in-game score and foul differentials.

#### 3.1.1. Game systems (non-home-and-away vs. home-and-away).

During the 2022–2023 season, the CBA operated under two competition systems: centralized non–home-and-away games and traditional home-and-away games. Under the non-home-and-away format, home and away teams won at nearly the same rate (home: 50.72%; away: 49.28%), a minimal difference of 1.44%. By contrast, under the home-and-away format, home teams won 65.22% of games while away teams won 34.78%, producing a much larger 30.43% difference. This comparison indicates that the two game systems constitute distinct competitive contexts: a clear home-court advantage is observed under the traditional home-and-away format, whereas such an advantage is not observed under the non-home-and-away format.

**Fig 1:**
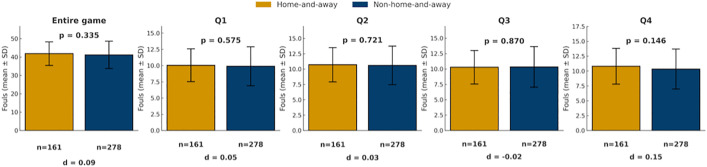
Comparison of foul calls between non-home-and-away and home-and-away systems. Bars show mean foul calls (± SD) for home teams (yellow) and away teams (blue) across the game and quarters. *p < 0.05, **p < 0.01, ***p < 0.001.

**Fig 2:**
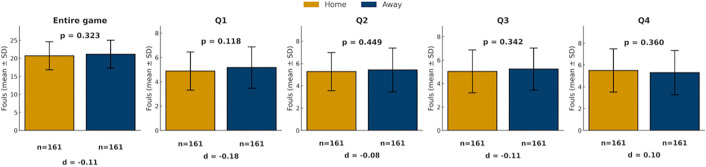
Comparison of foul calls between home and away teams in the home-and-away system. Bars show mean foul calls (± SD) for home teams (yellow) and away teams (blue) across the game and quarters. *p < 0.05, **p < 0.01, ***p < 0.001.

In terms of foul calls, however, no significant differences were found between the two systems in overall foul counts or in quarter-by-quarter comparisons (all p > 0.05; Fig 1). Within the subset of home-and-away games (Fig 2), home teams were called for slightly fewer fouls than away teams across the full game and in Q1–Q3, but these differences were not statistically significant (all p > 0.05).

#### 3.1.2. Game type (regular season vs. playoffs).

**Fig 3:**
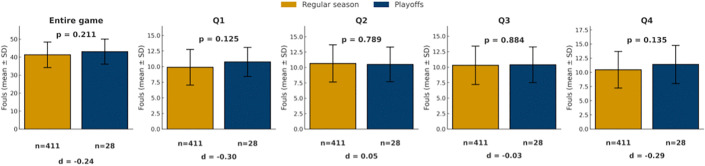
Comparison of foul calls between regular season and playoffs. Bars show mean foul calls (± SD) for regular season (yellow) and playoffs games (blue) across the game and quarters. *p < 0.05, **p < 0.01, ***p < 0.001.

Playoff games generally represent a higher intensity competitive context than regular season [31–33]. Despite this contrast, no significant differences in foul calls were observed between regular-season and playoff games, either at the game level or by quarter (all p > 0.05; Fig 3). At a descriptive level, playoff games tended to involve slightly more fouls overall, particularly in the first and fourth quarters, although these differences were not statistically significant.

Although foul counts did not differ significantly between regular season and playoffs, this finding should be interpreted with caution. The playoffs sample included far fewer games than the regular season sample, which may reduce statistical power in detecting game type difference. Although the event level dataset remained sufficiently large for model estimation, the imbalance at the game level may limit sensitivity to small differences between regular season and playoffs context.

#### 3.1.3. Team quality difference (large difference, small difference, evenly matched).

Games were categorized into three levels of team quality difference: evenly matched, small difference, and large difference, based on the clustering procedure described in the Methods (Table 2). This approach has been shown to reflect competitive balance more accurately than rank metrics [34].

**Table 2:** Levels of games by quality difference.

Games with Large Quality Difference (Differential of 2)	Games with Small Quality Difference (Differential of 1)	Evenly Matched Games (Differential of 0)
Weak vs. Strong (1 vs. 3)	Weak vs. Medium (1 vs. 2)	Weak vs. Weak (1 vs. 1)
Strong vs. Weak (3 vs. 1)	Medium vs. Weak (2 vs. 1)	Medium vs. Medium (2 vs. 2)
	Medium vs. Strong (2 vs. 3)	Strong vs. Strong (3 vs. 3)
	Strong vs. Medium (3 vs. 2)	

**Fig 4:**
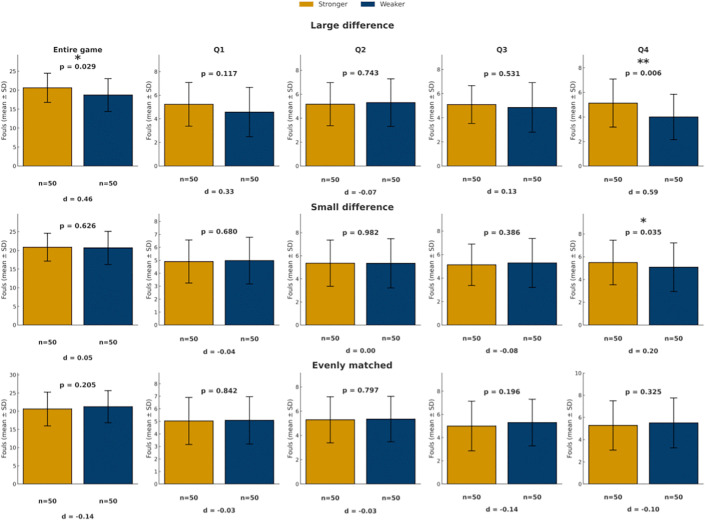
Comparison of foul calls under different team quality difference. Bars show mean foul calls (± SD) for stronger teams (yellow) and weaker teams (blue) across the game and quarters, categorized by game quality differences (large and small). In evenly matched games, colors indicate home (yellow) and away (blue) teams. *p < 0.05, **p < 0.01, ***p < 0.001.

As shown in Fig 4, in games with a large quality difference, strong teams received significantly more foul calls than weak teams overall (20.61 vs. 18.72 fouls, p < 0.05), with the effect concentrated in the fourth quarter (5.13 vs. 4.00 fouls, p < 0.01). In games with a small quality difference, no overall game-level differences were observed, although stronger teams received more fouls in the fourth quarter (p < 0.05). In evenly matched games, no significant foul calls differences between the two teams were found in any quarter or across the game as a whole (all p > 0.05).

Taken together, statistically significant difference in foul calls were mainly confined to games with larger team quality differences, particularly in the fourth quarter, while differences in other contexts were comparatively limited.

#### 3.1.4. Game conditions.

**Fig 5:**
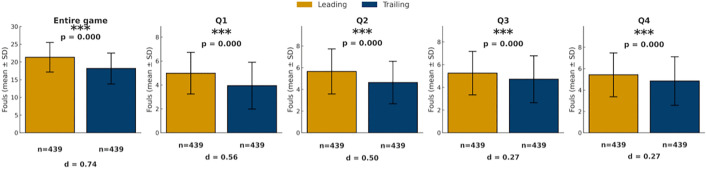
Comparison of foul calls between Leading and Trailing Teams. Bars show mean foul calls (± SD) for leading teams (yellow) and trailing teams (blue) across the game and quarters. *p < 0.05, **p < 0.01, ***p < 0.001.

3.1.4.1. *Score Differential (leading vs. trailing teams)*: Real-time score difference is one of the most important descriptors of game conditions. As shown in Fig 5, when there is a score differential, referees consistently called more fouls on the leading team than on the trailing team across the entire game and in each quarter (all p < 0.05).

**Fig 6:**
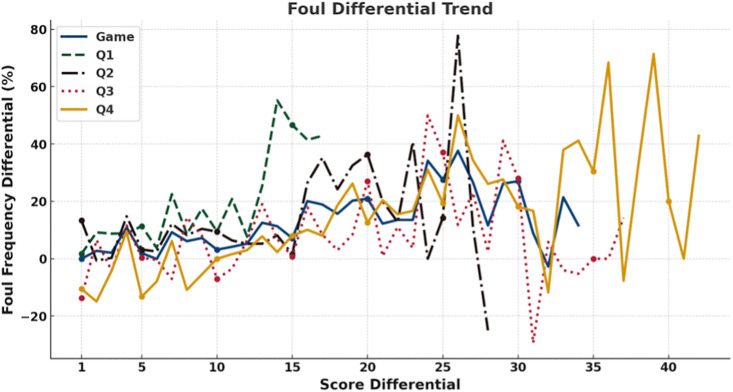
Trend of Changes in Foul Call Proportion Differences under Score Differential. Line plots show foul calls proportion differentials (y-axis) across score differentials (x-axis) for the entire game (blue) and for each quarter: Q1 (green), Q2 (black), Q3 (red), and Q4 (yellow).

To further illustrate the dynamic relationship, a line chart was employed (Fig 6), as the score differential is a continuous variable that changes throughout the game, making trends clearer than tables alone. At the game level, the proportion of fouls called on leading teams increased as the score gap widened, reaching approximately 20% higher than for trailing teams when the differential exceeded 20 points. At the quarter level, this pattern was most pronounced in the first quarter when the score gap was 10–15 points (leading teams were called for 47% more fouls) and in the second quarter when the gap exceeded 15 points (differential > 20%). By contrast, the fourth quarter showed no marked discrepancy in foul calls proportions between leading and trailing teams. Overall, the tendency for leading teams to receive more fouls strengthened as score differentials increased, particularly during the early and middle phases of games, while becoming less evident in the fourth quarter.

3.1.4.2 Foul Differential (more fouls vs. fewer fouls): Foul calls serve as a direct indicator of officiating decisions. Once a team was called more than four fouls in a quarter, all subsequent fouls (non-offensive foul) result in free throws for the opponent (bonus situation). Accordingly, foul differential was selected as a key variable to characterize game conditions, and was examined at both the game and quarter levels.

**Fig 7:**
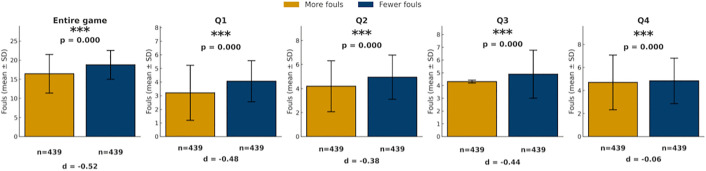
Comparison of foul calls between teams with more and fewer fouls (game). Bars show mean foul calls (± SD) for teams categorized as called more fouls (yellow) or fewer fouls (blue) across the game and quarters. *p < 0.05, **p < 0.01, ***p < 0.001.

As shown in Fig 7, there were significant differences in foul counts between teams with more fouls and teams with fewer fouls across the entire game and in each quarter (all p < 0.05), with teams holding fewer accumulated fouls consistently receiving more subsequent foul calls than those with more fouls.

**Fig 8:**
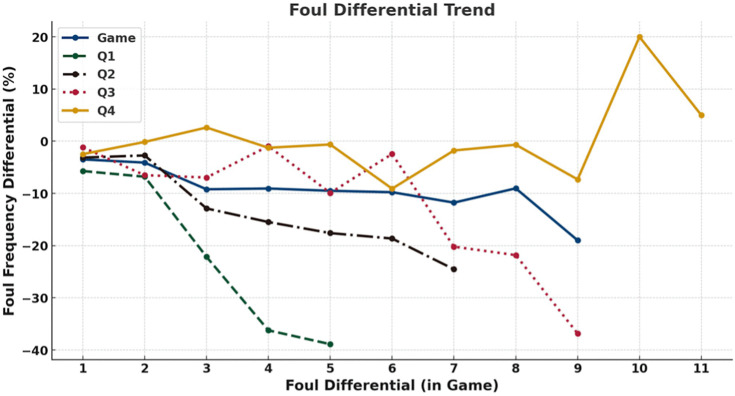
Trend of Changes in Foul Call Proportion Differences under Foul Differential (game). Line plots show foul calls proportion differentials (y-axis) across foul differentials within the foul differential in game (x-axis) for the entire game (blue) and for each quarter: Q1 (green), Q2 (black), Q3 (red), and Q4 (yellow).

Fig 8 further demonstrates that this tendency intensified as foul differential widened. As the foul differential increased, the proportion of subsequent fouls called on the team with more fouls decreased, while the proportion called on the team with fewer fouls increased. This tendency was most evident in the first quarter, where a five foul gap corresponded to the team with fewer fouls being called for 39% more fouls than the team with more fouls. In contrast, the fourth quarter showed a similar but less pronounced proportional difference trend.

**Fig 9:**
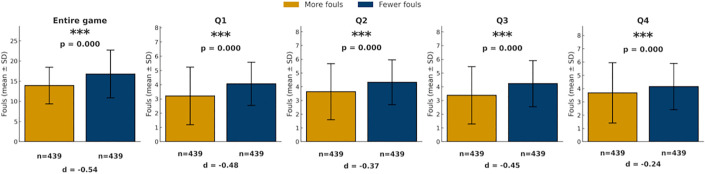
Comparison of foul calls between teams with more and fewer fouls (Quarter). Bars show mean foul calls (± SD) for teams categorized as called more fouls (yellow) or fewer fouls (blue) across the game and quarters. *p < 0.05, **p < 0.01, ***p < 0.001.

**Fig 10:**
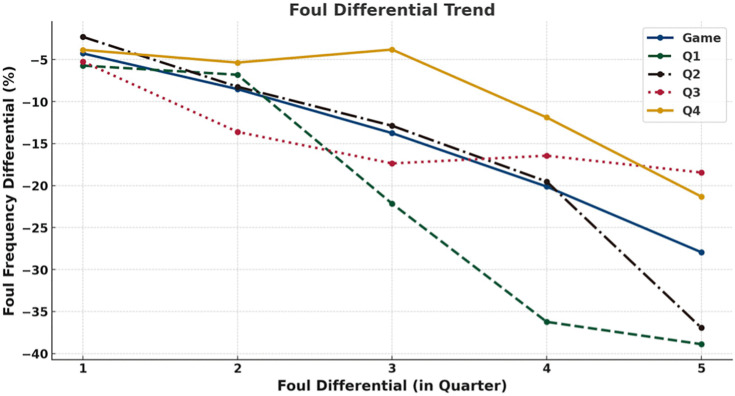
Trend of Changes in Foul Call Proportion Differences under Foul Differential (Quarter). Line plots show foul calls proportion differentials (y-axis) across foul differentials within the foul differential in quarters (x-axis) for the entire game (blue) and for each quarter: Q1 (green), Q2 (black), Q3 (red), and Q4 (yellow).

A quarter-level analysis yielded comparable findings (Figs 9 and 10). Across quarters, referees continued to call more fouls on teams with fewer fouls (all p < 0.05), with the difference most evident in the first quarter (4.06 vs. 3.21 fouls) and remaining significant in subsequent quarters. As the quarter-level foul gap increased, the difference in foul calls proportions between the two teams also increased: when the quarterly foul gap reached three or more fouls in the first three quarters, the team with fewer fouls was called for fouls more than 10% more than the team with more fouls. Although the fourth quarter showed a similar trend, the magnitude of the difference was smaller. When the quarter-level foul gap was between 1 and 4, the proportional differences between teams remained relatively small, only when the gap reached 5 did the team with fewer fouls receive a substantially higher proportion of fouls (21% more) than the team with more fouls.

Taken together, analyses of score and foul differentials reveal several consistent patterns across game conditions. First, when score differentials were present, fouls were more frequently called on leading teams, with this tendency generally intensifying as score gaps widened, particularly outside the fourth quarter. Second, across both game-level and quarter-level analyses, teams with fewer accumulated fouls systematically received more subsequent foul calls than teams with more fouls, and this tendency strengthened as foul differentials increased. Third, these patterns were most pronounced in earlier quarters and under larger differentials, whereas fourth-quarter effects were comparatively attenuated.

### 3.2. Logistic regression analysis of foul calls with multiple situational variables

To address Hypothesis 2, this section examines the combined associations of multiple situational variables with foul calls using multivariate logistic regression models across three comparative contexts. Binary logistic regression was applied to estimate the simultaneous influence of situational variables on foul calls, within a multivariate explanatory framework. The dependent variables were foul calls across three comparative contexts: home vs. away teams, leading vs. trailing teams, and teams with more vs. fewer fouls. These contexts were selected because they represent salient game situations and have been repeatedly identified in prior research as potential sources of referee bias.

#### 3.2.1. Logistic regression analysis of foul calls: Home vs. Away Teams.

**Fig 11:**
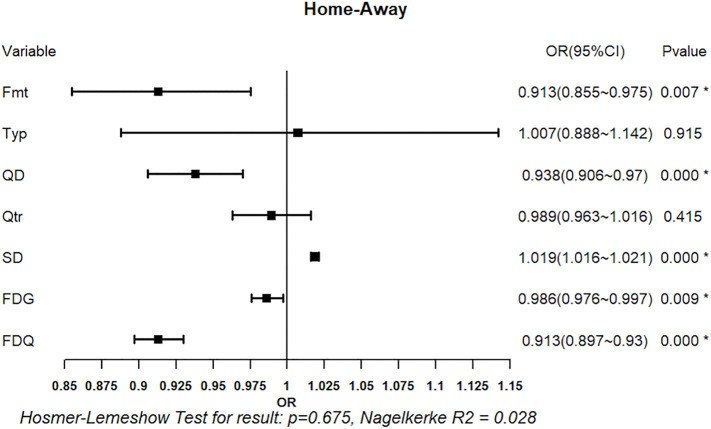
Regression Results of Situational Variables on Foul Calls (Home vs. Away Teams). Forest plot shows odds ratios (OR) with 95% confidence intervals (CI) for predictors of foul calls in home vs. away teams. Squares represent OR estimates and horizontal lines indicate 95% CIs. *p < 0.05.

Fig 11 presents the logistic regression results for foul calls on home versus away teams. The omnibus test of model coefficients indicated that the model was statistically significant (omnibus test, p < 0.05), and the Hosmer-Lemeshow test suggested acceptable model fit (p > 0.05). Among the explanatory variables, game systems XFmt (OR = 0.913, p < 0.05), team quality difference XQD (OR = 0.938, p < 0.05), score differential XSD (OR = 1.019, p < 0.05), game-level foul differential XFDG (OR = 0.986, p < 0.05), and quarter-level foul differential XFDQ (OR = 0.913, p < 0.05) were significant predictors of whether a foul was called on the home or away team. By contrast, game type XTyp and quarter XQtr were not significant (both p > 0.05).

It is noteworthy that although game systems was not significant in the single-factor analysis (Section 3.1.1), it emerged as significant in the regression model. This suggests that its effect may have been masked in simple comparisons but becomes evident when controlling for other situational variables.

#### 3.2.2. Logistic regression analysis of foul calls: Leading vs. Trailing teams.

**Fig 12:**
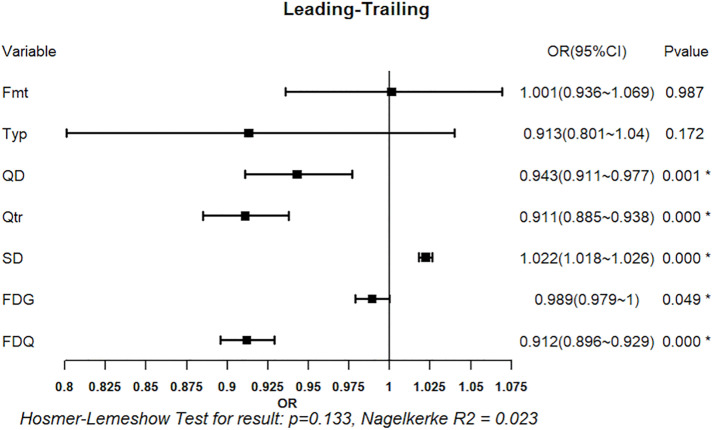
Regression Results of Situational Variables on Foul Call (Leading vs. Trailing Teams). Forest plot shows odds ratios (OR) with 95% confidence intervals (CI) for predictors of foul calls in leading vs. trailing Teams. Squares represent OR estimates and horizontal lines indicate 95% CIs. *p < 0.05.

The omnibus test again indicated that the overall model was statistically significant (p < 0.05), and the Hosmer-Lemeshow test supported good model fit (p > 0.05). Team quality differencel XQD, quarter XQtr, score differential XSD, game-level foul differential XFDG, and quarter-level foul differential XFDQ were all significant predictors (all p < 0.05), whereas game systems XFmt and game type XTyp were not significant (Fig 12).

#### 3.2.3. Logistic regression analysis of foul calls: More vs. Fewer Fouls.

**Fig 13:**
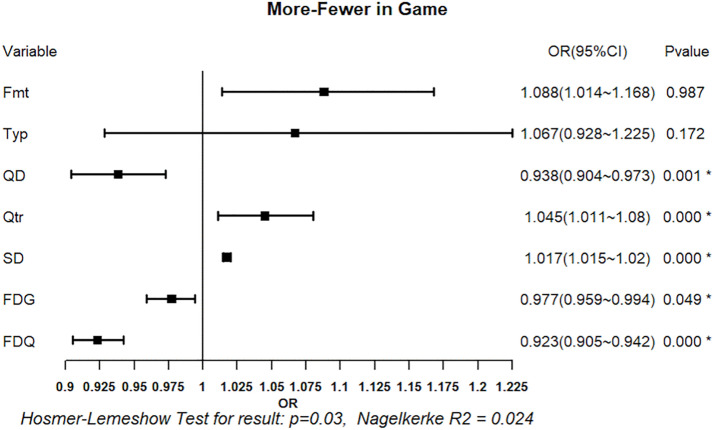
Regression Results of Situational Variables on Foul Call (More vs. Fewer Fouls in Game-Level). Forest plot shows odds ratios (OR) with 95% confidence intervals (CI) for predictors of foul calls in teams with more versus fewer fouls in a game. Squares represent OR estimates and horizontal lines indicate 95% CIs. *p < 0.05.

**Fig 14:**
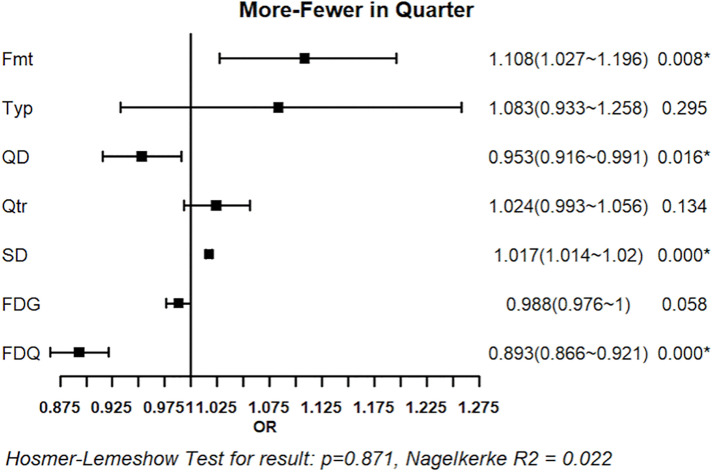
Regression Results of Situational Variables on Foul Call (More vs. Fewer Fouls in Quarter-level). Forest plot shows odds ratios (OR) with 95% confidence intervals (CI) for predictors of foul calls in teams with more versus fewer fouls in quarters. Squares represent OR estimates and horizontal lines indicate 95% CIs. *p < 0.05.

Figs 13 and 14 present the regression results for foul calls on teams with more versus fewer fouls, based on game-level and quarter-level foul differentials, respectively. Both models were statistically significant (omnibus test, p < 0.05). The game-level model, however, showed signs of poor fit (Hosmer–Lemeshow test, p < 0.05), whereas the quarter-level model demonstrated acceptable fit (p > 0.05). In the game-level model, team quality difference XQD (OR = 0.938, p < 0.05), quarter XQtr (OR = 1.045, p < 0.05), score differential XSD (OR = 1.017, p < 0.05), game-level foul differential XFDG (OR = 0.977, p < 0.05), and quarter-level foul differential XFDQ (OR = 0.923, p < 0.05) were significant variables, whereas game systems XFmt and game type XTyp were not. In the quarter-level model, game systems XFmt (OR = 1.108, p < 0.05), team-quality differential XQD (OR = 0.953, p < 0.05), score differential XSD (OR = 1.017, p < 0.05), and quarter-level foul differential XFDQ (OR = 0.893, p < 0.05) were significant, whereas game type XTyp, quarter XQtr, and game-level foul differential XFDG were not significant.

Across both models, team quality difference, score differential, and quarter-level foul differential consistently associated with foul calls. Stronger teams and leading team were more likely to be called, and teams with higher existing foul counts tended to receive relatively fewer additional fouls.

In all models, ORs indicate the direction of association between situational variables and the likelihood of a foul being called on the team coded as 1 in each comparative context. An OR greater than 1 reflects a higher likelihood of a foul being called on the home team, the leading team, or the team with more accumulated fouls, whereas an OR below 1 indicates a lower likelihood. Accordingly, the regression results are interpreted for explanatory rather than predictive purposes, with ORs serving as indicators of directional tendencies in foul calling rather than parameters intended to forecast foul-call outcomes.

## 4. Discussion

This study examined how multiple situational variables—including game systems, game type, team quality difference, score differential, and foul differential—shape foul calls in the CBA. Situated within the broader framework of sports performance analysis, the study extends the analytical focus from players and teams to referees, demonstrating that officiating performance can also be systematically evaluated through situational variables. Beyond documenting isolated effects, the present study explicitly examines how these situational factors operate when considered jointly, thereby providing a more comprehensive picture of how referees’ decisions are associated with contextual conditions. In contrast to prior research that has often isolated single factors, our analysis integrates both independent and combined associations within a unified analytical framework, offering insights that cannot be derived from single-factor comparisons alone. In doing so, the present study complements earlier work that has focused primarily on home advantage [9,10,27–30] or on specific types of calls such as technical or unsportsmanlike fouls [4,5,15], by revealing how multiple situational variables jointly shape foul-calling patterns at the event level in a professional league context.

The logistic regression models revealed that several of these variables, including game systems, became significant once other factors were controlled for. This multivariate finding highlights a key insight of the integrated situational approach adopted in this study: the absence of differences in single-factor analyses does not imply that a variable is irrelevant; rather, its effect may be masked by overlapping situational influences. Similar patterns have been reported in NCAA and NBA contexts [12,13]. By modeling situational variables simultaneously rather than in isolation, we show that foul calls are influenced by their simultaneous and overlapping effects, underscoring the importance of analyzing referees’ decisions within complex, dynamic game contexts.

One key result is the significant effect of the game systems once other variables are taken into account. Although the single-factor analysis did not reveal significant differences in foul counts between non–home-and-away and home-and-away formats, the logistic regression showed that the relative likelihood of fouls being called on home teams was reduced under the home-and-away format. This suggests that foul-calling patterns differed across game systems in more subtle ways that only become visible when contextual covariates are held constant. The CBA’s transition from centralized venues without spectators to traditional home-and-away games provides a natural experiment that is consistent with previous work on crowd effects and home advantage: when crowds are removed or attenuated, home bias tends to diminish, whereas the presence of spectators can introduce environmentally driven pressures on referees [22,35,36]. Our results extend these findings by showing that even when aggregate foul frequencies appear similar across systems, the probability of fouls being called on home versus away teams still varies once other situational variables such as score and foul differentials are accounted for, suggesting that environmental context and game condition jointly shape officiating. While crowd effects provide a theoretically grounded explanation, game-system differences may also reflect broader structural characteristics of competition formats, including scheduling intensity, travel and recovery demands, referee assignment, and organizational constraints.

Another important finding concerns foul differential. Across both game-level and quarter-level analysis, referees tended to call fouls on teams with fewer accumulated fouls, especially when foul gaps were large and in the early quarters. This supports the concept of a “balancing tendency” in officiating. Anderson and Pierce showed that NCAA referees were more likely to call fouls on the team with fewer fouls when the foul differential was large, and Price et al. reported similar balancing tendencies in the NBA [12,13]. Our results from CBA, showing that teams with fewer fouls became increasingly likely to be called as the foul gap widened—with differential in foul proportions exceeding 10–20% in some game and quarter contexts—are consistent in both direction and order of magnitude with these studies. Importantly, this tendency was not uniform across all game situations. Consistent effects were observed primarily when foul differentials were large and during the early and middle quarters, whereas the tendency was weaker in the fourth quarter and under smaller foul gaps, as reflected in both the single-factor analyses and the multivariate regression models. Taken together, these findings suggest that under comparable game conditions, foul balancing is not an idiosyncratic feature of a single competition, but a recurring pattern observed across different basketball cultures and governance systems [15].

The analysis of team quality differential and score differential showed that referees were more likely to call fouls against stronger or leading teams. This pattern should not be attributed solely to referees’ discretionary judgments, but also to teams’ tactical behaviors under specific game conditions. From a team perspective, stronger or leading teams often adopt situational strategies—such as increased defensive intensity, intentional fouling to stop fast breaks, or fouls taken to control tempo and manage rotations—particularly when protecting a lead [37–40]. These strategies inherently increase exposure to foul situations and may partly account for the higher foul rates observed among stronger or leading teams. At the same time, referees may be more inclined to impose stricter calls on stronger or leading teams in order to sustain competitive balance and preserve outcome uncertainty, which are central features of professional sport [41–43]. These findings highlight that observed foul-calling tendencies likely reflect an interaction between referees’ discretionary judgments and teams’ strategic behaviors, rather than officiating bias alone.

Beyond the statistical results, the findings carry important implications for understanding basketball officiating performance under different situational contexts. Referees are expected to act as neutral arbiters. However, the evidence suggests that their discretionary decisions are “context-sensitive” [44,45]. This raises the question of whether such tendencies should be interpreted as bias or as a functional adjustment aimed at sustaining perceived fairness and competitive balance. While balancing foul calls may reduce the appearance of unfairness, it could also inadvertently compromise true impartiality. For teams, recognizing such tendencies may aid tactical preparation—for example, being more cautious in foul-prone contexts or exploiting situations where referees tend to balance foul calls distributions.

Overall, the findings highlight that basketball officiating is situational rather than purely mechanical. Referees appear to strive for consistency, yet their decisions were associated with situational variables such as game system, foul difference, and score difference. For the CBA, this reveals both the professionalism of referees in maintaining stable standards and the subtle influence of situational variables that may affect officiating outcomes. More broadly, by embedding officiating within the sport performance analysis framework, the present study contributes to a growing body of work that treats referees as active performers whose decisions, like those of players and teams, are conditioned by dynamic game contexts. This perspective not only enriches academic understanding of officiating, but also offers practical guidance for league governance, referee training, and team strategy in professional basketball.

## 5. Limitations

This study has several limitations that should be acknowledged. First, foul calls arise primarily from players’ actual physical and tactical behaviors, which situational variables cannot fully capture. Our models focus on situational variables that show significant associations with foul-calling tendencies and, by design, cannot account for all foul outcomes or their fluctuations. Second, the analyses were based on situational variables commonly examined in sports performance analysis research. Although these variables reflect key aspects of game context, officiating behavior may also be influenced by additional information—such as player interactions, bench behavior, or media pressures—that was not available in the present dataset. Third, the study draws on data from a single CBA season with a specific combination of centralized non–home-and-away and home-and-away formats. While this setting provides a valuable quasi-experimental comparison across game systems, it may limit the generalizability of the findings to other seasons or leagues. In addition, broader structural features associated with competition formats were not explicitly modeled and may also contribute to observed game systems differences. Finally, the study focuses on objective situational variables but does not incorporate referees’ subjective judgment processes or potential sources of officiating bias. Future research could integrate psychological perspectives to better understand how situational variables and subjective factors jointly shape officiating decisions.

## 6. Conclusions and recommendations

This study investigated foul calls in the 2022–2023 CBA season under multiple situational variables. The findings indicate that although referees generally maintain relatively consistent officiating standards, foul calls are not fully independent of game situations. Stronger and leading teams were more likely to be called, whereas teams with fewer accumulated fouls were more likely to receive additional fouls, reflecting a “balancing” tendency. Logistic regression further highlighted team quality difference, score differential, and foul differentials as key situational variables influencing foul calls, whereas game type and competition format demonstrated more limited or conditional effects, becoming evident only when other situational variables were jointly considered.

The central contribution of this study is to show that referees’ decisions are systematically context-sensitive. These results underscore that officiating performance is inherently discretionary and shaped by situational variables. This situational characteristic can help sustain competitive balance and smooth game flow, but it also raises the risk of implicit bias in foul-calling. Recognizing this dual nature is essential for both referee training and team preparation.

By embedding officiating within the sport performance analysis framework, this study provides a structured approach to evaluating officiating performance. The findings contribute to the academic understanding of basketball officiating and also carry broader practical implications [46]. For leagues, recognizing the situational nature of officiating can inform competition management and policy-making, ensuring fairness and credibility in professional basketball. For referees, the results suggest that targeted training should focus on specific situations identified in this study—such as early-game contexts with large score or foul differentials—where balancing tendencies appear stronger. Scenario-based video review, structured feedback, and simulation training may help referees maintain consistency under these contextual pressures and mitigate unconscious bias [47–49]. For teams and coaches, understanding that foul-calling tendencies vary across game phases and differential magnitudes can support tactical preparation. In particular, teams may adopt more cautious defensive strategies when leading or anticipate balancing calls in games with large foul differentials. In training, players can be guided to better anticipate how situational factors may affect calls, helping them develop strategies to reduce unnecessary fouls or strategically use fouls when advantageous. Overall, the study enriches understanding of how situational variables shape refereeing and underscores their relevance not only for officiating evaluation, but also for league development, training design, and game strategy.
